# Recent Advance in Source, Property, Differentiation, and Applications of Infrapatellar Fat Pad Adipose-Derived Stem Cells

**DOI:** 10.1155/2020/2560174

**Published:** 2020-03-07

**Authors:** Yu-chen Zhong, Shi-chun Wang, Yin-he Han, Yu Wen

**Affiliations:** ^1^Department of Histology and Embryology, College of Basic Medical Sciences, China Medical University, Shenyang 110122, China; ^2^Class 4, Phase 102, China Medical University, Shenyang 110122, China

## Abstract

Infrapatellar fat pad (IPFP) can be easily obtained during knee surgery, which avoids the damage to patients for obtaining IPFP. Infrapatellar fat pad adipose-derived stem cells (IPFP-ASCs) are also called infrapatellar fat pad mesenchymal stem cells (IPFP-MSCs) because the morphology of IPFP-ASCs is similar to that of bone marrow mesenchymal stem cells (BM-MSCs). IPFP-ASCs are attracting more and more attention due to their characteristics suitable to regenerative medicine such as strong proliferation and differentiation, anti-inflammation, antiaging, secreting cytokines, multipotential capacity, and 3D culture. IPFP-ASCs can repair articular cartilage and relieve the pain caused by osteoarthritis, so most of IPFP-related review articles focus on osteoarthritis. This article reviews the anatomy and function of IPFP, as well as the discovery, amplification, multipotential capacity, and application of IPFP-ASCs in order to explain why IPFP-ASC is a superior stem cell source in regenerative medicine.

## 1. Introduction

In knee arthroplasty, infrapatellar fat pad (IPFP) as an inflamed tissue must be resected to relieve pain, so obtaining infrapatellar fat pad adipose-derived stem cells (IPFP-ASCs) avoids the secondary damage to patients as compared with obtaining mesenchymal stem cells (MSCs) derived from bone marrow, synovium, and subcutaneous fat. IPFP contains a large number of stem cells (5.5 × 10^6^ stem cells in 21 ml of IPFP), so more stem cells may be obtained from IPFP than from bone marrow [1].

Due to the presence of telomerase activity which can delay the aging process, IPFP-ASCs have strong proliferation capability and differentiation potential, namely, that IPFP-ASCs can be purified by multiple passages and then show stable differentiation potential [2]. Moreover, IPFP-ASCs are more superior in chondrogenic differentiation and osteogenic differentiation than bone marrow mesenchymal stem cells (BM-MSCs) [3] and synovial fluid mesenchymal stem cells (SF-MSCs) [4]. Due to IPFP anatomical position, the surface markers of IPFP-ASCs are similar to those of other cells around the knee joint, so IPFP-ASCs can be implanted in the knee joint with other synthetic grafts to reduce immunologic rejection [5]. IPFP-ASC is a good seed cell for autologous or allogenic stem cell transplantation because of its strong proliferation capability and weak immunologic rejection [6, 7].

IPFP, a subsidiary tissue to protect and support the knee joint, may have repair effects on the knee joint by secreting cytokines [8]. Currently, it is difficult for osteoarthritis to repair knee joint tissue and control disease progression due to weak regeneration capability and reduced blood supply [5], so the treatment for osteoarthritis focuses on relieving pain and protecting remaining joint function. In recent years, it is reported that autologous stem cell transplantation has a certain therapeutic effect on osteoarthritis [9]; SF-MSCs can repair cartilage, mesenchymal stem cells (MSCs) can relieve inflammatory pain by secreting prostaglandin E2 (PGE2) regulating the expression of inflammatory factors in surrounding cells, and the injection of supernatant fluid after IPFP-ASC culture into the knee joint can relieve inflammatory pain and restore partial joint function [10]. Therefore, IPFP has both physical protection effect and repair effect provided by paracrine secretion on the knee joint.

Scaffolds are usually used to improve stem cell molding growth. Compared with BM-MSCs and subcutaneous adipose-derived stem cells (Sc-ASCs), IPFP-ASCs grow better in the environment with scaffolds [11]. It is reported that IPFP-ASCs have a good application future in 3D bioprinting [12]. Therefore, IPFP-ASCs are more suitable to molding culture.

IPFP-ASC has become a focus in regenerative medicine and tissue engineering because IPFP-ASCs can be differentiated into bone, cartilage, and fat. Therefore, we reviewed the anatomy and function of IPFP, as well as the discovery, amplification, multipotential capacity, and application of IPFP-ASCs in order to explain why the IPFP-ASC is a high-quality source of stem cells for regenerative medicine. The review article provides a reference for clinical surgical treatment and regenerative medicine.

## 2. Anatomical Structure and Physiological Function of IPFP

IPFP, yellowish adipose tissue, is located among the patellar tendon, femoral condyle, and tibial plateau with a triangular shape. The tip of the IPFP is attached to the front of the intercondylar fossa, the bottom of the IPFP is below the basis patellae and on both sides of the patellar tendon, and the partial bottom is between the two-layer synovium. IPFP is an inverted triangle in shape, and the two angles of IPFP are located on both sides of the patellar tendon; the inner side of IPFP exceeds the medial margin of the patellar ligament about 2.5 cm, and the outer side of IPFP exceeds the lateral margin of the patellar ligament about 2.4 cm; IPFP stretches about 2 cm upward and about 1 cm outward, and IPFP finally is closely attached to the patellar medial inferior and lateral inferior sides, respectively [13]. In knee bend, IPFP enters the joint cavity to fill the empty space; while in strong contraction of the quadriceps femoris, the IPFP plays a role in preventing knee joint hypermobility. However, once IPFP hardens, IPFP may grow into the joint cavity and cause the limitation of joint motion. The blood supply of IPFP is mainly from the arteria genu inferior medialis and arteria genu inferior lateralis.

The main physiological function of IPFP is to reduce friction and protect knee joint during joint motion. IPFP can prevent patellar injury by stabilizing patella and reducing friction [14]. Takatoku et al. [15] have found that IPFP is very important for patellar formation and development in immature rabbits. Besides cushioning shock and reducing friction, IPFP has a fixed effect on joint movement. IPFP also has repair effects on the injured knee joint because IPFP-ASCs in IPFP can differentiate into cartilage and can secrete cytokines such as platelet-derived growth factor (PDGF), vascular endothelial growth factor (VEGF), and fibroblast growth factor (FGF), which play antiapoptotic roles along with transforming growth factor-*β* (TGF-*β*).

## 3. Comparison of IPFP-ASCs with Other MSCs

Since Zuk et al. [16] discovered and cultured processed lipoaspirate (PLA) cells in 2001, a great deal of attention has been paid to adipose-derived stem cells (ASCs). In 2003, Wickham et al. [17] isolated stromal cells which can be differentiated into bone, cartilage, and fat from IPFP for the first time, providing a basis for investigating MSCs. The application of MSCs in tissue repair has become a hot issue in regenerative medicine and has attracted a great deal of attention from many research studies. Therefore, it is very important to choose a superior stem cell source. MSCs are mainly derived from bone marrow, synovium, subcutaneous adipose tissue, and IPFP. The MSCs derived from various sources are different in differentiation potential, proliferation rate, and surface markers. The surface markers are similar between IPFP-ASCs and BM-MSCs, but different surface markers between IPFP-ASCs and BM-MSCs are also present in some samples, which may be related to the different physiological status of samples and different culture conditions of stem cells.

Garcia et al. [4] have reported that the proliferation capability of SF-MSCs is weaker than that of IPFP-ASCs. Compared with Sc-ASCs, the differentiation capability is stronger and the cell volume is smaller in IPFP-ASCs [12, 18]. CD34 and CD45 expression levels are higher in IPFP-ASCs than in BM-MSCs; and cartilage gene expression levels, especially SOX9 and COL2A1, are more active in IPFP-ASCs than in Sc-ASCs and Wharton's jelly stem cells (WJSCs) [5]. The above studies suggest that IPFP-ASC has marked advantages in proliferation and differentiation, especially in chondrogenic differentiation, and its strong proliferation capability provides a basis for subsequent large-scale studies. Culture efficiency and surface markers of MSCs from different sources are shown in Table 1.

### 3.1. Differentiation Potential of MSCs from Different Sources Associated with the Growth Microenvironment and the Paracrine of Surrounding Cells

There is a lot of dense connective tissue around IPFP; but around Sc-ASCs, there is a great deal of subcutaneous tissue. The different growth microenvironments and paracrine of surrounding cells influence their proliferation capability and differentiation potential [26]. IPFP-ASCs can maintain stable chondrogenic differentiation potential during proliferation [3]. In addition, different cytokines, culture methods, and growth microenvironments are associated with IPFP-ASC differentiation potential, which will be introduced in detail in Section 4.3 of this article.

### 3.2. Proliferative Stability of MSCs from Different Sources Probably Determined by the Integrity of Telomere

Telomere is a nucleoprotein located in the terminal end of the eukaryotic chromosome, and its length is regarded as a biomarker of aging. The maintenance of telomerase activity suggests that the cells have antiaging ability [27]. Until the 18th passage, IPFP-ASCs still have a growth rate and adipogenic differentiation potential which is confirmed by peroxisome proliferator-activated receptor *γ*2 and lipoprotein lipase [28]. This may be that IPFP-ASCs possess telomerase activity and telomere integrity, which allow IPFP-ASCs to have mitotic potential after multiple passages [2]. These findings prove IPFP-ASC proliferation capability and also provide an idea for future studies.

### 3.3. Effects of Different Physiological Conditions on Proliferation Capability and Differentiation Potential of MSCs

In different physiological conditions, IPFP-ASCs have different proliferation capability and differentiation potential. In some knee joint diseases such as localized nodular synovitis, Hoffa's disease, or arthrofibrosis, the physiological function of MSCs from IPFP with different involvement levels is various. Local anesthetics can weaken the adipogenic differentiation potential of IPFP-ASCs [29]. The chondrogenic differentiation potential of IPFP-ASCs is stronger in obese donors than in thin donors [30]. The osteogenic differentiation potential of IPFP-ASCs is stronger in rheumatoid arthritis than in osteoarthritis, which may be related to tissue necrosis factor-*α* (TNF-*α*) [31]. Osteoarthritis can affect the chondrogenic differentiation potential of IPFP-ASCs [32]. The decreased chondrogenic differentiation potential of IPFP-ASCs may be associated with the downregulation of *TGF-β* gene expression in osteoarthritis [33]. It remains to be further investigated whether the IPFP-ASCs with stronger chondrogenic differentiation potential can be obtained by gene knockout.

### 3.4. Various Differentiation Potential and Proliferation Capability of MSCs from Bodily Different Parts

Oxygen tension can promote the proliferation of BM-MSCs and IPFP-MSCs [34]. Suprapatellar fat pad adipose-derived stem cells (SPFP-ASCs) can specifically express CD105; SPFP-ASCs have stronger chondrogenic differentiation potential and better effects on relieving the symptoms of osteoarthritis in rat models than IPFP-ASCs [35]. Adipose-derived stem cells isolated from lipoaspirates have stronger chondrogenic differentiation potential as compared with IPFP-ASCs [36]. Amaral et al. [37] have reported that cartilage cells differentiated by SF-MSCs are more stable and functional than those differentiated by IPFP-ASCs in vitro; but after subcutaneous injection of the two kinds of stem cells, there is no significant difference in differentiation potential between SF-MSCs and IPFP-ASCs in vivo. Above studies suggest that besides growth microenvironment, stem cell differentiation potential and proliferation capability are also related to stem cell sources and culture methods. In order to understand their interactions, it is necessary to further study the physiological process of stem cell proliferation.

## 4. Isolation, Culture, Identification, and Differentiation Potential of IPFP-ASCs

### 4.1. Isolation and Culture of IPFP-ASCs

Obtaining IPFP-ASCs requires a series of steps, including separation, mechanical digestion, chemical digestion, purification, and plastic attachment. The process includes shearing, collagenase digestion, impurity removal, filtration, centrifugation, supernatant removal, and transfer culture. The usage of collagenase is not uniform. It has been reported that usage of 0.075% collagenase I requires 2 h for digestion [38] and 0.1% collagenase I combined with one percentage of bovine serum containing CaCl_2_ requires one hour for digestion [9], while there also is the usage of 0.25% trypsin which requires 20 min for digestion [31]. One study indicated that if 0.2% collagenase I was used for 10 min, the vascular matrix component could be obtained and if the 0.2% collagenase I was used for more than 30 min, the number of live adipocytes was reduced [39]. The addition of trypsin to collagenase I can accelerate the enzyme digestion. In future research, it is necessary to select the best type and concentration of enzyme to isolate stem cells quickly and efficiently.

The common Sc-ASCs are derived from the liquid fat of subcutaneous tissue, so the liquid-dispersed morphology is conducive to obtaining stem cells; while IPFP-ASCs are derived from the solid fat in IPFP, so the solid fat must undergo physical shearing and chemical digestion into liquid fat, which may affect the time to obtain stem cells and the quantity and proliferation capability of stem cells. Recent studies have shown that human second passage IPFP-ASC into the third passage IPFP-ASC requires approximately 48-60 h [26, 36, 38]. The total number of cells has a certain effect on cell proliferation capability, which may be related to intercellular communication and microenvironment [40, 41]. We should pay attention to these factors in order to obtain more stem cells with strong proliferation capacity. Francis et al. [39] found some optimized isolation methods such as frequent stirring, increasing collagenase, and using cell-specific coating flasks, which could obtain MSCs within 85 min and greatly shorten the time to obtain MSCs as compared with the previous 24-48 h. This makes it possible for time to use IPFP-ASCs in tissue transplantation of surgical emergencies. However, real application of IPFP-ASCs in acute trauma and transplantation surgery still requires a large number of clinical studies.

The culture conditions of IPFP-MSCs are similar to those of ordinary MSCs and commonly include 37°C, 5% CO_2_, and saturated humidity. After primary culture, trypsin digestion is used for serial subcultivation.

### 4.2. Identification of IPFP-ASCs

There are many methods for the identification of stem cells. At present, the common identification methods include histology, immunohistochemical staining, qPCR, biochemical analysis, and image and mechanical detection, among which, the most commonly used method is immunohistochemical staining.

In 2006, the International Society for Cellular Therapy (ISCT) gave the definition of multipotential stromal cells and the bases to distinguish the multipotential stromal cells from hematopoietic stem cells as follows: (1) cell adhesion growth in vitro; (2) positive expression of CD44, CD73, CD90, and CD105 (positive detection rate of flow cytometry > 95%) and negative expression of CD11b, CD14, CD19, CD34, CD45, CD79*α*, and HLA-DR (the positive detection rate of flow cytometry < 2%); and (3) having the capabilities to differentiate into osteoblasts, adipocytes, and cartilage cells in vitro in specific culture conditions. IPFP-ASCs are not good for staining using low-affinity nerve growth factor receptors or STRO-1 [42]. In addition, IPFP-ASCs have higher expression levels of CD34, CD45, and CD166 than BM-MSCs, which suggests that IPFP-ASCs are different from BM-MSCs, but both still have similar surface markers such as CD73, CD90, and CD105 [26]. In the posttenth passage IPFP-ASC surface, there is the expression of CD13, CD44, CD90, and CD105 and occasionally the expression of 3G5, but no expression of LNGFR, STRO-1, CD34, and CD56 [28]. In the third to fifth passage IPFP-ASCs, the expression of CD13, CD45, and CD43 is negative and the expression of CD29, CD44, and CD90 is positive [43]. A systematic study on IPFP-ASCs shows that the surface antigens of IPFP-ASCs are CD9+, CD10+, CD13+, CD29+, CD44+, CD49e+, CD59+, CD105+, CD106+, and CD166+ [21]. So far, no specific markers have been found during IPFP-ASC culture, which may be related to the coexistence of multiple differentiated directional cells, so it is necessary to screen the unique target cells [26].

### 4.3. Differentiation Potential of IPFP-ASCs

MSC directional differentiation has always been a hot topic. The studies on inducing IPFP-ASC differentiation are mainly about chondrogenic differentiation and secondly about osteogenic differentiation and adipogenic differentiation. Certainly, there are a few studies on MSC differentiation to other directions. This article gives the introduction one by one as follows.

#### 4.3.1. Chondrogenic Differentiation

At present, there are three main methods to induce chondrogenic differentiation including (1) regulation of physical factors for culture medium, (2) utilization of paracrine and intercellular interaction by coculture with other cells, and (3) differentiation-related gene transfection. However, it has been unclear which factor has stronger effect on differentiation and what interactions are present among the three factors. These issues remain to be further investigated.

Adjusting the inorganic culture environment by changes of biophysical factors may obtain different culture efficacies. Hydrostatic pressure plays an important role in IPFP-ASC chondrogenic differentiation [34]. Intermittent hydrostatic pressure may have a stronger effect on the chondrogenic differentiation of cartilage progenitor cells than on the chondrogenic differentiation of IPFP-ASCs [44]. Hypoxia (5%) is conducive to IPFP-ASC chondrogenic differentiation but can decrease IPFP-ASC osteogenic differentiation in mice [45]. Hyaluronic acid is an important extracellular matrix for chondrogenesis. The culture medium containing 25% hyaluronic acid is very conducive to IPFP-ASC chondrogenic differentiation, but culture medium containing 25% to 75% hyaluronic acid has no effect on the proliferation capability of IPFP-ASCs [5]. The cartilage similar to the structure and function of natural cartilage can be obtained by controlling the oxygen concentration of hydrogel and the surrounding environment [46]. The chondrocyte-laden hydrogels supported by a superficial layer of stem cells allow MSCs to have stronger chondrogenic differentiation potential, suggesting that the orderly environment is more conducive to MSC differentiation [38]. The recent studies on chondrogenic differentiation of MSCs and IPFP-ASCs are shown in Table 2.

Because IPFP is near the synovium and cartilage, IPFP-ASCs in IPFP may be induced to differentiate into chondrocytes by the growth factor secreted by its surrounding cells [21]. An in vivo 3D culture of IPFP-ASCs indicates that the cartilage matrix scaffold treated by TGF-*β*3 and the gelatum treated by TGF-*β*1 promote IPFP-ASC chondrogenic differentiation [57, 58]. The coculture of IPFP-ASCs with articular cartilage cells and hyaluronic acid can promote IPFP-ASC chondrogenic differentiation and prevent the occurrence of hypertrophic articular cartilage [48]. Above studies suggest that there are close communications between cells and the surrounding environment.

In addition, there are studies on IPFP-ASC differentiation at gene level. Bravo et al. [32] have found that during IPFP-ASC chondrogenic differentiation, the expressions of PTFR and MMP13 are similar between the patients with knee osteoarthritis and the individuals with healthy knees, but the expressions of OPG, FGF2, TGF, and MMP3 are significantly lower in the patients with knee osteoarthritis than in the individuals with healthy knees.

#### 4.3.2. Osteogenic Differentiation

Osteogenic differentiation is also an important research direction of IPFP-ASCs. Compared with chondrogenic differentiation, the studies about osteogenic differentiation mainly focus on gene regulation and external scaffold construction, but there are also a few studies about cytokines. Osteogenic differentiation potential is stronger in the IPFP-ASCs from obese mice than in the IPFP-ASCs from emaciated mice [67]. Osteogenic capability of IPFP-ASCs is not significantly different from BM-MSCs [3] and SF-MSCs [4]. IPFP-ASCs only produce bone precursor protein when induced by the culture medium containing vitamin C and inorganic phosphorus, but bone formation protein-2 (BMP-2) can induce IPFP-ASC osteogenic differentiation [1]. After coculture of fetal cartilage with IPFP-ASCs on polycaprolactone scaffold, the expressions of type II collagen and polyproteoglycan significantly decrease, but the expression of Indian hedgehog factor significantly increases, suggesting that children cartilage and polycaprolactone have strong osteogenic effect and weak chondrogenic effect on IPFP-ASCs [68]. The recent studies on osteogenic differentiation of ASCs and IPFP-ASCs are shown in Table 3.

#### 4.3.3. Adipogenic Differentiation

Due to limited utilization of differentiated adipocytes, adipogenic differentiation is not the main research direction of IPFP-ASCs, but it is still an important part of MSCs. Adipogenic differentiation potential is stronger in IPFP-ASCs than in BM-MSCs [3] and SF-MSCs [4]. Obese mice have stronger IPFP-ASC adipogenic differentiation potential than thin mice [67]. Hyaluronic acid also can enhance adipogenic differentiation [52]. Lopa et al. [84] have reported that IPFP-ASC adipogenic differentiation potential is similar between young people and old people. This is different from that IPFP-ASC chondrogenic differentiation potential is stronger in young people than in old people, which remains to be further explored.  Table 4 shows the articles on the adipogenic differentiation-related factors.

#### 4.3.4. Inducing Differentiation toward Other Sorts of Body Cells

At present, there are no reports on IPFP-ASC differentiation into other sorts of body cells besides chondrogenic, osteogenic, or adipogenic differentiation. However, MSCs may be differentiated into multilineage cells. MSCs have the potential to differentiate into osteoclasts [90]. MSCs also show cardiomyocyte-like, skeletal muscle cell-like, tendon-like, and epithelial cell-like differentiation [91, 92]. Canine MSCs can be immortalized with SV40 gene, and then the immortalized MSCs are transplanted into busulfan-induced seminiferous tubules of infertile mice and show germ cell differentiation potential [93]. The studies on differentiation toward other histiocytes are shown in Table 5.

## 5. Applications of IPFP-ASCs and MSCs

IPFP-ASCs are generally considered to be more promising stem cells because IPFP as an inflamed tissue must be resected to relieve pain in knee arthroplasty, so obtaining IPFP-ASCs avoids the damage to patients as compared with obtaining bone marrow-derived MSCs, and IPFP-ASC isolation procedure is easy to operate. The injection of IPFP-ASCs can effectively relieve pain and improve knee joint function in osteoarthritis [100, 101]. Koh et al. [100, 101] reported that after injection of platelet-rich plasma containing a mean of 1.89 × 10^6^ IPFP-ASCs into arthritic knees, the mean Lysholm, Tegner activity scale, and VAS scores improved significantly and no major adverse events related to the injections were observed; the therapeutic effects were positively associated with the number of IPFP-ASCs but negatively associated with disease duration and patient's age. IPFP-ASCs show marked therapeutic effects in young patients and patients with early cartilage deformation [98, 99]. The chondrogenic differentiation potential of IPFP-ASCs from the patients with advanced OA is decreased [102]. IPFP-ASCs have good effects on osteoarthritis in stages I and II [103]. IPFP-ASCs may be induced to produce muscle-specific cytoskeleton proteins which are conducive to muscle regeneration [9].

Besides subpatellar fat pad, MSCs are also obtained from subgluteal fat pad, abdominal liposuction, and inguinal fat pads. MSCs from these different sources all show good functional characteristics. The postoperative evaluation is better in BM-MSC transplantation combined with microfracture than in microfracture alone [104]. The outcomes of subgluteal fat pad-derived MSC implantation for cartilage repair of osteoarthritic knees seem encouraging [105]. The graft survival and the volumes of fat and newly generated connective tissue all are significantly higher in abdominal liposuction-derived MSC-enriched fat grafts than in fat grafts alone, suggesting that MSC-enriched graft could render lipofilling a reliable alternative to major tissue augmentation, such as breast surgery or major flap surgery [106]. A combination of percutaneously injected autologous adipose tissue-derived stem cells, hyaluronic acid, platelet-rich plasma, and calcium chloride may regenerate bone and cartilage in the patients with hip osteonecrosis or knee osteoarthritis [107]. The applications of IPFP-ASCs and MSCs are shown in Table 6.

At present, most studies on IPFP-ASC are about in vitro IPFP-ASC culture and IPFP-ASC animal experiment. In recent years, 3D printing technology has solved the nonmolding problem of stem cell differentiation and can construct complex human structure and improve cell differentiation efficiency and/or posttransplantation survival ability. Stem cells may be differentiated into cartilage by microfluidics without the requirement of growth factors [12]. With the development of stem cell technology and biomaterial science, IPFP-ASCs will be more and more widely used.

## 6. Summary and Outlook

Compared with other MSCs, IPFP-ASCs have the following characteristics: (1) IPFP-ASC isolation is relatively easy, and more stem cells may be obtained from the IPFP. (2) IPFP-ASCs have strong proliferation capability and differentiation potential, and IPFP-ASCs are more superior in chondrogenic differentiation and osteogenic differentiation than BM-MSCs and Sc-ASCs. (3) IPFP-ASCs can secrete a variety of cytokines. The injection of IPFP-ASC culture supernatant fluid into the knee joint can relieve inflammatory pain and restore partial joint function, which is related to miR-100-5p-abundant exosomes produced by IPFP-ASCs in the supernatant fluid [115]. This demonstrates that IPFP-ASCs have broad prospects in the treatment of knee osteoarthritis. (4) IPFP-ASCs may be used in treatment of osteoarthritis along with other methods. Due to IPFP anatomical position, the surface markers of IPFP-ASCs are similar to those of other cells around the knee joint, so IPFP-ASCs can be implanted in the knee joint with other synthetic grafts to reduce immunologic rejection. (5) IPFP-ASCs are more suitable to molding culture. (6) Other aspects include the following. IPFP-ASCs have immunomodulatory effect. IPFP-ASCs as adult stem cells also are used in the reconstruction of various tissues.

In summary, many problems on IPFP-ASCs remain to be solved, but more and more attention has been paid to it because IPFP collection is easy, IPFP contains a large number of stem cells, IPFP-ASC isolation is simple, and IPFP-ASCs have multidirectional differentiation potential. IPFP-ASCs will be a promising stem cell source in regenerative medicine after constantly overcoming the existing problems such as optimizing pretreatment stem cell immune specificity and homogeneity, improving IPFP-ASC differentiation capacity into target cells, exploring the best transplantation stage, and increasing posttransplantation regeneration rate. We believe that the key problem resolved in the future is that the methods for IPFP-ASC directional differentiation will be safe, controllable, and manipulable.

## Figures and Tables

**Table 1:** Culture efficiency and surface markers of IPFP-ASCs and other MSCs.

Sources	Culture efficiency	Surface markers	References
Marrow	Strong differentiation potential and low culture efficiency in BM-MSCs	CD13+, CD29+, CD44+, CD49a–, f+, CD51+, CD73+, CD90+, CD105+, CD106+, CD166+, STRO-1+, CD11b-, CD14-, CD19- CD34-, CD45-	[19]
IPFP	Higher culture efficiency in IPFP-ASCs	CD13+, CD29+, CD44+, CD90+, CD105+, CD34-, CD56-, CD27-1-, STRO-1-	[20, 21]
Synovium	Stronger chondrogenic differentiation in SF-MSCs than in IPFP-ASCs	CD44+, CD90+, CD105+, CD147+, CD34-, CD45-, CD117-, CD31-	[22, 23]
Subcutaneous fat	Stronger differentiation potential in Sc-ASCs than in BM-MSCs	CD29+, CD44+, CD90+, CD13+, CD105+, CD34-, CD45-, CD31-, CD133-	[24, 25]

IPFP-ASCs: infrapatellar fat pad adipose-derived stem cells; MSCs: mesenchymal stem cells; BM-MSCs: bone marrow mesenchymal stem cells; IPFP: infrapatellar fat pad; Sc-ASCs: subcutaneous adipose-derived stem cells.

**Table 2:** Factors associated with chondrogenic differentiation of IPFP-ASCs and MSCs.

Year	Species	Cells	Factors	Outcome	Reference
2019	Sheep	IPFP-ASCs	Nanofiber polycaprolactone	Promoting chondrogenic differentiation	[47]
2018	Human	BM-MSCs	Microfluidic model technology	Inducing chondrogenic differentiation	[12]
2018	Human	IPFP-ASCs	Gelatin scaffolds with aligned holes or random holes	Better chondrogenic differentiation in the gelatin scaffolds with aligned holes than in the gelatin scaffolds with random holes	[11]
2018	Human	IPFP-ASCs	Coculture with platelet-rich plasma	Failing to promote chondrogenic differentiation	[48]
2018	Human	ASCs from Lonza (Basel, Switzerland)	Second passage ASCs treated using endothelin-1	Unfavourable for chondrogenic differentiation	[49]
2018, 2012	Human	IPFP-ASCs	Coculture with articular chondrocytes in hypoxia (5%)	Promoting chondrogenic differentiation	[50, 51]
2017	Human	IPFP-ASCs	Coculture with hyaluronic acid nanoparticles	Promoting chondrogenic differentiation and preventing articular cartilage thickening and inflammation	[52]
2017	Pig	BM-MSCs	Culture with different material scaffolds in hypoxia (5%)	Hypoxia enhances cell viability and the expression of chondrogenic markers, and cellular response is superior with polycaprolactone than with hyaluronic acid	[53]
2017	Human	IPFP-ASCs	Indirect coculture with osteoarthritis-derived articular chondrocytes	Promoting chondritic phenotypic recovery and IPFP-ASC chondrogenic differentiation in osteoarthritis	[54]
2017	Human	IPFP-ASCs	Culture of ascorbic acid-treated IPFP-ASCs	The hardness of matrix is unchanged, but the chondrogenic differentiation potential is enhanced	[55]
2017	Human	IPFP-ASCs and Sc-ASCs	The culture medium containing TGF-*β* family-related growth factors	Promoting chondrogenic differentiation	[36]
2017	Human	IPFP-ASCs	Knocking out RHEB	Decreasing chondrogenic and osteogenic differentiation	[56]
2016	Human	IPFP-ASCs	Porous cartilage extracellular matrix stent containing TGF-*β*3	Continuously promoting chondrogenic differentiation	[57]
2017	Human	IPFP-ASCs	Injectable hydrogel containing TGF-*β*1 and H_2_O_2_	H_2_O_2_ allows the hydrogels to create a high-pressure environment which combined with TGF-*β*1 continuously promotes chondrogenic differentiation	[58]
2016	Pig	IPFP-ASCs	Poly(*ε*-caprolactone) membrane	Inducing stem cells to form cartilage matrix, which enhances chondrogenic differentiation	[59]
2016	Sheep	IPFP-ASCs	Culture of IPFP-ASCs under low-intensity pulsed ultrasound	Increased expression of cartilage gene	[60]
2016	Human	IPFP-ASCs	Coculture with rat chondrocytes and acellular dermal matrix	Promoting cartilage formation and infiltration	[61]
2016	Human	IPFP-ASCs	Coculture with osteoarthritis-derived articular chondrocytes	Failing to promote chondrogenic differentiation	[62]
2015	Pig	IPFP-ASCs	Culture of IPFP-ASCs under dynamic pressure	Dynamic pressure slightly promotes chondrogenic differentiation and is conducive to structural stability	[63]
2015	Human	IPFP-ASCs	Implantation of CD44 and IPFP-ASCs into TGF-*β*3 eluting ECM-derived stents	Producing more sulfated glycosaminoglycan and type II collagen, which promotes chondrogenic differentiation	[64]
2011	Bear	Sc-ASCs	Pellet culture	Promoting chondrogenic differentiation	[65]
2011	Cattle	IPFP-ASCs	The 3^rd^ to 12^th^ passage IPFP-ASCs	Unfavourable for chondrogenic differentiation	[7]
2009	Human	BM-MSCs	Culture of BM-MSCs with dexamethasone	Promoting chondrogenic differentiation by enhancing expression of cartilage extracellular matrix genes	[66]
2003	Human	IPFP-ASCs	Embedded with fibrin glue followed by culture in cartilage medium	Occurrence of chondrogenic differentiation after 6-week culture	[1]

ASCs: adipose-derived stem cells; MSCs: mesenchymal stem cells; IPFP-ASCs: infrapatellar fat pad adipose-derived stem cells; BM-MSCs: bone marrow mesenchymal stem cells; Sc-ASCs: subcutaneous adipose-derived stem cells; RHEB: ras homolog enriched in brain; TGF-*β*3: transforming growth factor-*β*3; ECM: extracellular matrix.

**Table 3:** Factors associated with osteogenic differentiation of ASCs and IPFP-ASCs.

Year	Species	Cells	Factors	Outcome	Reference
2018	Human	Sc-ASCs (epididymal fat pads)	Knocking out *FAK* gene	Promoting osteogenic differentiation	[69]
2018	Human	Sc-ASCs	AtECM	Reducing the activity in early osteogenesis	[70]
2017	Human	IPFP-ASCs	Coculture with fetal cartilage on polycaprolactone scaffold	Reduced type II collagen and polyproteoglycan and increased Indian hedgehog factor	[68]
2017	Human	IPFP-ASCs	RHEB overexpression in IPFP-ASCs	Promoting osteogenic differentiation	[56]
2016	Human	ASCs from Life Technology (USA)	MSCs cultured in 20%, 40%, and 60% FB scaffolds	Promoting osteogenic differentiation, and osteogenic differentiation is more obvious in 40% and 60% FB scaffolds	[71]
2016	Human	ASCs from Life Technology (USA)	Scaffolds with large stiffness and small aperture	Osteogenesis is preferential	[71]
2016	Mice	Sc-ASCs (inguinal fat pads)	The 3^rd^ to 12^th^ passage Sc-ASCs	Increased osteogenic differentiation	[6]
2016	Mice	Sc-ASCs (inguinal fat pads)	Low-density sowing	Toward osteogenic differentiation	[6]
2015	Human	Sc-ASCs	Osteogenic induction medium containing GLP-1	Promoting osteogenic differentiation	[72]
2014	Rat	Sc-ASCs (inguinal fat pad)	Medium containing rhPDGF-BB	Promoting osteogenic differentiation	[73]
2014	Human	Sc-ASCs	Amplification on BM-ECM	Producing more osteoblasts	[74]
2014	Human	Sc-ASCs	Subcutaneous implantation of MSCs induced by BM-ECM in nude mice	Producing more bone tissue	[74]
2014	Human	ASCs from Lonza (Basel, Switzerland)	Addition of fullerol	Improving osteogenic potential	[75]
2013	Mice	IPFP-ASCs	Obesity	Promoting osteogenesis	[67]
2012	Mice	Sc-ASCs	Inhibiting *zfp467* gene	Stimulating osteoblast development	[76]
2012	Rat	Sc-ASCs (inguinal fat pads)	Mechanical load or maintaining static stretching	Promoting osteogenesis	[77]
2011	Human	Sc-ASCs	*Noggin* gene knockout	Enhancing osteogenic differentiation	[78]
2011	Human	Sc-ASCs	BMP-2	Enhancing osteogenic potential	[78]
2011	Bear	Sc-ASCs	Bone growth factor	Promoting osteogenesis	[65]
2011	Cattle	IPFP-ASCs	Multiple passages	Toward osteogenic differentiation	[7]
2010	Mice	ASCs (inguinal fat pads)	shh-N overexpression in ASCs	Promoting osteogenic differentiation	[79]
2009	Mice	Sc-ASCs (inguinal fat pads)	Inhibition of histone deacetylase activity in reduced oxygen environment	Promoting osteogenic differentiation	[80]
2008	Pig	Sc-ASCs	rhBMP-6 overexpression in Sc-ASCs	Inducing bone formation and obtaining spinal fusion	[81]
2006	Rat	Sc-ASCs (inguinal fat pads)	Runx-2 overexpression in Sc-ASCs	Enhancing osteoblastic differentiation	[82]
2003	Human	IPFP-ASCs	BMP-2 cDNA overexpression in IPFP-ASCs	Promoting osteogenesis	[1]
2002	Rat	BM-MSCs	Fluid flow	Increasing mineralized matrix deposition in a dose-dependent manner	[83]

ASCs: adipose-derived stem cells; IPFP-ASCs: infrapatellar fat pad adipose-derived stem cells; Sc-ASCs: subcutaneous adipose-derived stem cells; AtECM: adipose tissue extracellular matrix; RHEB: ras homolog enriched in brain; FB: firming buffer; GLP-1: glucagon-like peptide-1; rhPDGF-BB: recombinant human platelet-derived growth factor; BM-ECM: bone marrow-extracellular matrix; BMP-2: bone morphogenetic protein-2; shh-N: N-terminal Sonic Hedgehog; rhBMP-6: recombinant human bone morphogenetic protein-6.

**Table 4:** Factors associated with adipogenic differentiation of ASCs and IPFP-ASCs.

Year	Species	Cells	Factors	Outcome	Reference
2018	Human	ASCs from Lonza (Basel, Switzerland)	Treated with ET-1	Promoting adipogenic differentiation	[50]
2018	Human	ASCs from Lonza (Basel, Switzerland)	Inducing ASCs after inhibiting ET receptor	Promoting adipogenic differentiation	[50]
2018	Human	Sc-ASCs	AtECM	Promoting adipogenic differentiation in the first 7 days of differentiation induction	[70]
2017	Human	IPFP-ASCs	Hyaluronic acid	Promoting adipogenic differentiation	[52]
2017	Human	IPFP-ASCs	RHEB overexpression in IPFP-ASCs	Promoting adipogenic differentiation	[56]
2017	Human	Sc-ASCs	Addition of 2, 5, and 10 *μ*m of pyrintegin	Lipid accumulation is the most marked in 2 *μ*m of pyrintegin	[85]
2016	Human	Sc-ASCs	miRNA-29b overexpression in Sc-ASCs	Promoting adipogenic differentiation	[86]
2016	Human	ASCs from Life Technology (USA)	ASCs cultured in 20%, 40%, and 60% FB scaffolds	Promoting adipogenic differentiation, and osteogenic differentiation is the most obvious in 20% FB scaffolds	[71]
2016	Human	ASCs from Life Technology (USA)	Scaffolds with low stiffness and large aperture	Promoting adipogenic differentiation	[71]
2016	Mice	Sc-ASCs (inguinal fat pads)	The 3^rd^ to 12^th^ passage Sc-ASCs	Inhibiting adipogenic differentiation	[6]
2015	Human	Sc-ASCs	Adipogenic induction medium containing GLP-1	Inhibiting adipogenic differentiation	[72]
2014	Rat	Sc-ASCs (inguinal fat pads)	ERK inhibitor (PD98059)	Promoting adipogenic differentiation	[73]
2014	Mice	Sc-ASCs (inguinal fat pads)	Removing ovaries	Promoting adipogenic differentiation	[87]
2013	Mice	IPFP-ASCs	Obesity	Promoting adipogenic differentiation	[67]
2013	Rat	Sc-ASCs (inguinal fat pads)	Secretory factors from rat adipose tissue explants	Promoting adipogenic differentiation	[88]
2012	Mice	Sc-ASCs	Inhibiting *zfp467* gene	Inhibiting adipogenic differentiation	[76]
2012	Pig	Sc-ASCs	A differentiation inducer which consisted by dexamethasone, insulin, and methylxanthine and combined with myostatin	Inhibiting adipogenic differentiation	[89]
2012	Rat	Sc-ASCs (inguinal fat pads)	Mechanical cyclic loading on Sc-ASCs	Inhibiting adipogenic differentiation	[77]
2011	Bear	Sc-ASCs	Adipogenic induction medium	Lipid droplet accumulation	[65]
2011	Cattle	IPFP-ASCs	The fifth passage	Inhibiting adipogenic differentiation	[7]
2010	Mice	Sc-ASCs (inguinal fat pads)	Blocking endogenous hedgehog signaling pathway and adding hedgehog antagonists	Promoting adipogenic differentiation	[79]
2009	Mice	ASCs (inguinal fat pads)	Transient exposure to histone deacetylase inhibitor under hypoxia	Inhibiting adipogenic differentiation	[80]
2006	Rat	Sc-ASCs (inguinal fat pads)	Runx-2 overexpression in Sc-ASCs	Inhibiting adipogenic differentiation	[82]

ASCs: adipose-derived stem cells; IPFP-ASCs: infrapatellar fat pad adipose-derived stem cells; ET-1: endothelin-1; AtECM: adipose tissue extracellular matrix; RHEB: ras homolog enriched in brain; FB: firming buffer; GLP-1: glucagon-like peptide-1; ERK: extracellular signal-related kinase.

**Table 5:** Differentiation potential toward other histiocytes.

Year	Species	Cells	Differentiation direction	Reference
2018	Human	Sc-ASCs	Skeletal muscle cells	[94]
2014	Human	Sc-ASCs	Sc-ASCs promote the proliferation of vascular endothelial cells with the aid of VEGF-A	[95]
2013	Rat	ASCs (inguinal fat pads)	Vascular endothelial cells and tubular cells	[88]
2008	Rat	ASCs (inguinal fat pads)	ASCs attached to the polyurethane can significantly increase VEGF level, and the ASCs implanted subcutaneously in rats can increase the microvessel density of the surrounding tissue	[96]
2006	Human	Sc-ASCs	Pancreatic endocrine cells	[97]
2005	Human	Sc-ASCs	Hepatocytes	[98]
2002	Human/mice	Sc-ASCs	Nerve cells	[99]

IPFP-ASCs: infrapatellar fat pad adipose-derived stem cells; ASCs: adipose-derived stem cells; Sc-ASCs: subcutaneous adipose-derived stem cells; VEGF-A: vascular endothelial growth factor A.

**Table 6:** Applications of IPFP-ASCs and MSCs.

Year	Species	Diseases	Cells	Methods	Outcome	Reference
2018	Human	Osteoarthritis	Ha-MSCs	Injection of Ha-MSCs into knee joint cavity	No severe side effects, relieving pain, restoring function, increasing cartilage mass	[108]
2018	Human	Osteoarthritis	Sc-ASCs	Injection of Sc-ASCs into knee joint cavity	Reducing cartilage defects by regeneration of hyaline-like articular cartilage	[109]
2018	Human	Osteoarthritis	Sc-ASCs	Injection of PRG into knee joint cavity	Relieving pain	[11]
2018	Human	In vitro model of meniscal tear	Sc-MSCs	Coculture medium of Sc-MSCs and TGF-*β* was added into bovine meniscus tear in vitro model	Showing strong matrix-sulfated proteoglycan deposition and promoting healing	[110]
2016	Human	Osteoarthritis	Sc-ASCs	Injection of Sc-ASCs (2 × 10^6^, 10 × 10^6^, and 50 × 10^6^, respectively) into knee joint cavity	Relieving pain and restoring function, especially in 2 × 10^6^ Sc-ASCs	[111]
2015	Human	Bone destruction of long bone	Sc-ASCs	Transplantation of three-dimensional graft from autologous Sc-ASCs and decalcified bone matrix into knee joint	Promoting osteogenesis in bone nonunions and restoring anatomic structure without oncological side effects	[112]
2014	Rabbit	Osteoporosis	Sc-ASCs	Injection of Sc-ASCs into knee joint cavity	Promoting bone regeneration in vivo and relieving osteoporosis	[113]
2014	Rat	Abdominal aortic aneurysm	Sc-ASCs	Injection of Sc-ASCs into abdominal aortic aneurysm	Promoting the expression of elastin in smooth muscle and facilitating the reconstruction of elastic membrane	[114]
2012	Human	Osteoarthritis	IPFP-ASCs	Injection of IPFP-ASCs into knee joint cavity	Effectively relieve pain and improve the function of knee joint	[99]
2011	Human	Calvarial defects	Sc-ASCs	Transplantation of Sc-ASCs with *Noggin* knockout into the destroyed skull in mice	Rapidly repairing mouse calvarial defects	[78]
2010	Mice	Tibia destruction	Sc-ASCs	Transplantation of ASCs stimulated by N-terminal sonic hedgehog into tibial defect	Enhancing osteogenesis	[79]
2008	Pig	Lumbar disc degeneration	IPFP-ASCs	Injection of IPFP-ASCs with rhBMP-6 overexpression into lumbar paravertebral muscle	Spinal fusion and new bone formation	[81]
2003	Human	Osteoarthritis	IPFP-ASCs	IPFP-ASCs were embedded into fibrin glue nodules, for inducing chondrogenic cells, and were placed in osteogenic media containing BMP-2 for inducing osteogenic cells, respectively. And then these cells were transplanted into the hind legs of nude mice	Cartilage formation induced by fibrin glue nodules, bone formation induced by BMP-2, and bone marrow cavity formation in the hind legs of the osteogenic culture group	[1]

IPFP-ASCs: infrapatellar fat pad adipose-derived stem cells; MSCs: mesenchymal stem cells; Ha-MSCs: human amniotic mesenchymal stromal cells; Progenza (PRG): comprises in vitro expanded mesenchymal stem cells derived from human donor adipose tissue combined with cell culture supernatant; TGF-*β*: transforming growth factor-*β*; rhBMP-6: recombinant human bone morphogenetic protein-6; BMP-2: bone morphogenetic protein-2.
